# Supplementary material for: Research on the generative logic and configuration effects of the policy implementation environment in China’s grassroots digital construction: Traceability based on grounded theory and the validation of the csQCA method
Source: PLoS One. 2024 Apr 18;19(4):e0301667. doi: 10.1371/journal.pone.0301667 (PMC11025859; doi:10.1371/journal.pone.0301667)
Supplement: Attachment — Submitted filename: Response to Reviewers.doc [file pone.0301667.s001.doc]

**Response to Reviewers**

Dear review experts, thank you for reviewing our manuscript.

According to your review opinions, we have made a comprehensive revision. The contents of the revision mainly include: first, the introduction part of the manuscript has been comprehensively revised, especially for the revision opinions of the review experts; Second, the second part of the manuscript has been modified, focusing on the source selection of literature, data collection and big data management issues, and adding a paragraph of discussion; The third is to make targeted changes to the table, diagram, formatting and participating documents according to the standard of this journal; Fourthly, we hired MDPI professionals to make native language modifications to the use of language in our manuscript, so that our manuscript could achieve native language discussion.

For details of the changes, see the marked document. Please review it.

Salute to you!

Liu bangfan

2024/2/21
